# Gene expression profiles in genome instability-based classes of colorectal cancer

**DOI:** 10.1186/s12885-018-5174-z

**Published:** 2018-12-18

**Authors:** Vincenza Barresi, Giacomo Cinnirella, Giovanna Valenti, Giorgia Spampinato, Nicolò Musso, Sergio Castorina, Daniele F. Condorelli

**Affiliations:** 1 Department of Biomedical and Biotechnological Sciences, Section of Medical Biochemistry, University of Catania, Viale Santa Sofia 89-97, 95123 Catania, Italy; 2 Laboratory of Complex Systems, Scuola Superiore di Catania, University of Catania, Catania, Italy; 3 Department of Surgical Medical Sciences and Advanced Technologies “G. F. Ingrassia”, University of Catania, Catania, Italy; 4 Fondazione Mediterranea G.B. Morgagni, Catania, Italy

**Keywords:** Colorectal cancer, Broad copy number aberrations, Gene expression profiles, Consensus molecular subtypes, Mucinous colorectal tumors

## Abstract

**Background:**

Broad copy number aberrations (BCNAs) represent a common form of genome instability in colorectal cancer (CRC). CRCs show large variations in their level of aneuploidy: microsatellite-instable (MSI) tumors are known to have a near-diploid karyotype while microsatellite-stable (MSS) tumors show high level of chromosomal instability. However, MSS tumors have great heterogeneity in the number of BCNAs, with a minor percentage of samples showing an almost normal karyotype. In the present work we subdivided MSS CRCs according to a “BCNA score” and characterized their transcriptome profiles, considered as a proxy to their phenotypic features.

**Methods:**

Microsatellite testing, genome-wide DNA copy number and whole-transcript expression analysis (HTA) were performed on 33 tumor samples and 25 normal colonic tissue samples from 32 CRC patients. 15.1% of the samples were MSI tumors (*n* = 5), whereas 84.9% were MSS tumors (*n* = 28). Gene expression data of 34 additional MSI tumors was retrieved from a public functional genomics data repository.

**Results:**

Using as a threshold the first quartile of the BCNA score distribution, MSS samples were classified as low-BCNA (LB, *n* = 7) or high-BCNA (HB, *n* = 21). LB tumors were enriched for mucinous CRCs and their gene-expression profile resembled that of MSI samples for what concerns a subset of genes involved in secretory processes, mucosal protection, and extracellular matrix remodeling. HB tumors were predominantly non-mucinous adenocarcinomas and showed overexpression of a subset of genes typical of surface colonocytes and EGF signaling. A large percentage of unclassified samples according to the consensus molecular subtypes (CMS) classifier was found in the LB group (43%), whereas 76% HB tumors belonged to CMS2.

**Conclusions:**

A classification of colorectal tumors based on the number of BCNAs identifies two groups of MSS tumors which differ for histopathology and gene expression profile. Such information can be exploited for its translational relevance in different aspects of CRC clinical management.

**Electronic supplementary material:**

The online version of this article (10.1186/s12885-018-5174-z) contains supplementary material, which is available to authorized users.

## Background

Colorectal cancer (CRC) can be characterized by different forms of genome or epigenome instability, encompassing chromosomal instability (CIN), microsatellite instability (MSI), CpG island methylator phenotype (CIMP) and high single nucleotide mutation rates (hypermutation-ultramutation) [1].

Cancer cells with CIN tend to acquire chromosomal abnormalities, namely gains or losses of chromosomes or sub-chromosomal portions, at a rate higher than normal along cell divisions [2]. Since measuring CIN as the rate of acquisition of chromosomal changes is difficult in solid tumors, the amount of copy number aberrations (CNAs) is used as a surrogate marker. Indeed, the frequency of somatic CNAs is dependent on length in a biphasic way: the most abundant classes of CNAs are represented by small-size CNAs (focal CNAs) and large-size CNAs (length of chromosome arm or whole chromosome) [3, 4]. Broad copy number aberrations (BCNAs) can be defined as copy number abnormalities involving a large percentage of a chromosomal arm or an entire chromosome [3–6]. BCNAs are better identified in comparison to focal CNAs of smaller size, even in the presence of tumor heterogeneity or admixtures of tumor and normal cells [5]. Therefore, it has been proposed to focus on BCNAs for CIN-based classification of tumors [5].

Microsatellite instability is a well-known form of genome instability caused by a defective mismatch repair system, with tumor cells being unable to keep a constant length of repetitive microsatellite sequences scattered throughout the genome [7]. MSI tumors are known to have a near-diploid karyotype [8–11]. On the other hand, among microsatellite-stable (MSS) tumors, two groups can be distinguished according to the amount of chromosomal aberrations: tumors with low numbers of BCNAs and tumors with high numbers of BCNAs. It is possible that the presence of a high or low amount of BCNAs translates into phenotypic differences in cancer cells, and transcriptomic profile might represent a proxy to such phenotypic features [12].

The aim of the present work was to characterize the gene expression profiles of these two groups of MSS tumors, and compare them to the transcriptional phenotype of MSI tumors.

## Methods

### Sample cohort

Thirty-three tumor samples (clinicopathological data in Additional file 1: Table S1) were collected from a cohort of 32 patients who underwent resection of primary invasive CRC at “Centro Clinico Diagnostico S.r.l. G.B. Morgagni” in Catania (Italy). All patients gave informed consent for this study, which was approved by the Ethics Committee of ASL3 of Catania (Italy). All specimens were frozen and stored at − 80 °C until DNA and RNA extraction. In one case, a synchronous tumor located in another site of the colon was present and biopsied. The number of individual tumors, taking into account the patient with two synchronous tumors (considered as separated entities) was of 33. A biopsy of adjacent phenotypically normal colonic tissue (at a distance of 3–6 cm from the tumor) was taken for 25 patients (tumor/normal pairs).

### Microsatellite testing

Samples were tested for microsatellite instability with five markers belonging to the Bethesda panel (D2S123, D5S346, D17S250, BAT25 and BAT26) and one additional marker (BAT40) [5]. Tumors were defined as MSI if ≥30% markers were found unstable when comparing tumor versus normal colonic tissue. 5/32 (15.6%) patients had MSI tumors, whereas 27/32 (84.4%) patients had MSS tumors.

### Genome-wide DNA copy number and SNP genotyping analysis

Genome-wide DNA copy number and SNP genotyping analysis were performed in 33 tumor samples and 25 normal tissue samples on Affymetrix SNP 6.0 arrays (Affymetrix, Inc., Santa Clara, CA, USA), using 500 ng of input DNA. Array scanning and data analysis were performed by Affymetrix® “GeneChip Command Console” (AGCC) and “Genotyping Console™” (GTC) version 3.0.1 software [13, 14] . Broad copy number abnormalities (BCNAs), defined as gains or losses involving more than 25% of a chromosomal arm or numerical aberrations involving whole chromosomes, were identified by using a bioinformatic tool called BroCyA, as described by Barresi et al. (2017). Briefly, an estimate of copy number for each DNA marker in each sample was obtained by calculating the log2ratio between fluorescent intensity signal in the sample and the corresponding median value in the reference group composed of 270 HapMap individuals. Log2ratio of each DNA marker is calibrated assuming the reference group value equal to 2 (diploid genome). DNA markers in autosomes and in female X chromosomes with calibrated log2ratios greater than 2.21 (average+ 2 S.D. of a control normal diploid group) have been considered as gains and markers with values less than 1.74 (average – 2S.D.) as losses. For the X and Y male chromosomes 1.24 and 0.81 were used as upper and lower limit, respectively. The algorithm searched all short segments, formed by at least 50 contiguous markers showing the same type of copy number variation (short isosegments) and then joined them in larger segments (BCNAs) and retained them in the final list if the following conditions were met: 1) their intersegment distance is lower than an established threshold [5], 2) the entire BCNA (short isosegments and intersegment intervals) has a mean calibrated log2ratio higher than 2.21 or lower than 1.74 for broad gains and losses, respectively, 3) the entire BCNA has a physical size higher than 25% of a chromosomal arm. Finally, a score was attributed to each chromosomal arm or to whole chromosome according to the following rules:

- the score “1” was attributed to each chromosomal arm (p or q) if BCNAs are present only in that chromosomal arm or in the q arm of an acrocentric chromosome.
- if BCNAs of the same type (gains or losses) are observed on both p and q arms and their sum expressed as “%p + %q” is greater than 150 a score of “1” was attributed to the whole chromosome (w);
- if BCNAs are observed on both p and q arms and their sum expressed as “%p + %q” is less than 150 the score “1” was attributed to both p and q arms.

The sum of the scores per tumor sample corresponds to the “BCNA score”.

The logic behind our BCNA scoring system (p-q-w system) is to evaluate as a single mutational event (with a score of 1) the lost or the gain of a whole chromosome. For instance, in the p-q-w scoring system “n” segmental aneuploidies get a “BCNA score” equal to that of “n” whole chromosome aberrations. We are aware that other scoring system systems count p and q aberrations as separate events, even in the case of whole chromosome aberrations. We also recalculated BCNA scores according to a p-q scoring system (attributing a score of 2 to a whole chromosome aberration). Although the absolute values of BCNA scores are different in the two systems the subdivision of MSS tumors in two classes, as reported in the result section, is not affected by the choice of the scoring system.

Raw and processed data of SNP 6.0-array results have been submitted to public repository: “Gene Expression Omnibus-GEO” (www.ncbi.nlm.nih.gov/geo) with the following accession number: GSE80460 [5]. Data of the most frequent DNA copy number changes in a cohort of 27 MSI samples were available from a study by Sveen et al. (2017) [15].

### Whole-transcript expression analysis

Whole-transcript expression analysis was performed from 100 ng of total RNA by amplification and target hybridization to the Gene-Chip Human Transcriptome Array (HTA) 2.0 (Affymetrix, Inc., Santa Clara, CA, USA), as previously described [16]. Array scanning and data analysis were performed by Affymetrix® Expression Console™ software version 1.4 and Affymetrix® Transcriptome Analysis Console (TAC v3) software (Affymetrix, Inc., Santa Clara, CA, USA). Transcript level analysis was performed using the normalization method based on the processing algorithm called robust multi-array average (RMA). Such RMA values are log_2_ values. Average RMA values have been transformed in linear values and their ratios (linear fold changes) have been used in order to estimate differential expression between CRC groups and normal colon group. Fold changes < 1 have been reported as the negative of the reciprocal, so that e.g. a fold change of ½ is reported as − 2. Therefore, linear fold-changes (denominated FC in the rest of the text) were calculated in the following way: 2^[CRC group Average RMA – Normal Colon group average RMA]^ if CRC group > Normal Colon group, or − 2^[Normal Colon group Average RMA – CRC group average RMA]^ if CRC group < Normal Colon group. Statistical analysis of differential gene expression was performed as implemented in the TAC software using one-way ANOVA analysis and *p*-value correction for multiple testing according to Benjamini-Hochberg [17].

The data have been deposited to public repository: ‘Gene Expression Omnibus-GEO’ (www.ncbi.nlm.nih.gov/geo) and are accessible through GEO: GSE73360 [16] and GSE84984.

Since in our series of 33 tumor samples there were only 5 MSI tumors, we did not include such samples for the expression analysis (TAC software), and used instead genome-wide HTA data of 34 MSI samples from Sveen et al. (2017) [15], deposited by the authors on the NCBI Gene Expression Omnibus (GEO) with the accession number GSE79959.

When analyzing whole-transcript HTA data, we ruled out genes with no gene symbol assigned by Affymetrix, genes which had a gene symbol of the type “OTTHUMG###”, genes described by Affymetrix as “uncharacterized LOC” (except for those with more than one description, the first one only beginning with “uncharacterized LOC”), genes encoding small nucleolar RNAs (snoRNAs), small Cajal body-specific RNAs (scaRNAs), small nuclear RNAs (snRNAs), small NF90-associated RNAs, RNA 5S ribosomal genes and pseudogenes, RNA 5.8S ribosomal pseudogenes, Y RNAs, mitochondrially encoded ribosomal RNAs (MT-RNR), microRNAs, olfactory receptor and histone cluster genes. We also did not consider transcripts on chr1_gl000191_random, chr4_ctg9_hap1, chr4_gl000193_random, chr4_gl000194_random, chr6_apd_hap1, chr6_cox_hap2, chr6_dbb_hap3, chr6_mann_hap4, chr6_mcf_hap5, chr6_qbl_hap6, chr6_ssto_hap7, chr7_gl000195_random, chr17_ctg5_hap1, chr17_gl000204_random, chr19_gl000209_random, chrUn_gl000211, chrUn_gl000212, chrUn_gl000218, chrUn_gl000219, chrUn_gl000220, chrUn_gl000222, chrUn_gl000223, chrUn_gl000228.

### Consensus molecular subtype classifier (CMSclassifier)

In their paper on the consensus molecular subtypes (CMS) of colorectal cancer, Guinney et al. (2015) [12] provided a downloadable R package (CMSclassifier), which included the Random Forest classifier and the Single Sample Predictor (SSP) classifier. SSP gives a prediction of the CMS of a tumor sample regardless of whether it is analyzed alone or within a series of samples. Our input data were formatted as requested by the software instructions (https://github.com/Sage-Bionetworks/CMSclassifier/blob/master/README.md) and included 5969 of the 5973 genes of the example data set. The 4 missing genes were due to the fact that in the transcript cluster (TC)-to-Entrez ID conversion table provided by Affymetrix NetAffx™ Analysis Center website (https://www.affymetrix.com/), 4 TCs had two Entrez IDs each, and both the IDs were included in the example data set of the CMSclassifier R package as unique entries. In particular, in the Affymetrix conversion table, IDs 701 and 56,924 were both assigned to TC15000276.hg.1; IDs 6038 and 283 to TC14000067.hg.1; IDs 6560 and 3931 to TC16001199.hg.1; IDs 54,741 and 3953 to TC01000730.hg.1. Therefore, IDs 701, 6038, 6560, 54,741 were ruled out, in order to provide a unique Entrez ID for each transcript cluster.

## Results

### Genome instability (GI)-based classification

MSS CRCs were subdivided in two groups according to the distribution of the BCNA scores: a low-BCNA (LB) group bearing a BCNA score lower than the first quartile (5.75) of the distribution (7 tumor samples from 7 patients with 0–5 BCNAs per tumor) and a high-BCNA (HB) group, including all the other MSS tumors (21 tumor samples from 20 patients with 6–21 BCNAs per tumor). The average BCNA score for the LB samples was 1.85 (SD = 2.03, *n* = 7) whereas for the HB group it was 12.76 (SD = 4.65, *n* = 21). BCNA scores of our series of MSI tumors were lower than the first quartile of MSS BCNA distribution, and had an average count equal to 2.6 (SD = 1.67, *n* = 5).

**Fig. 1:**
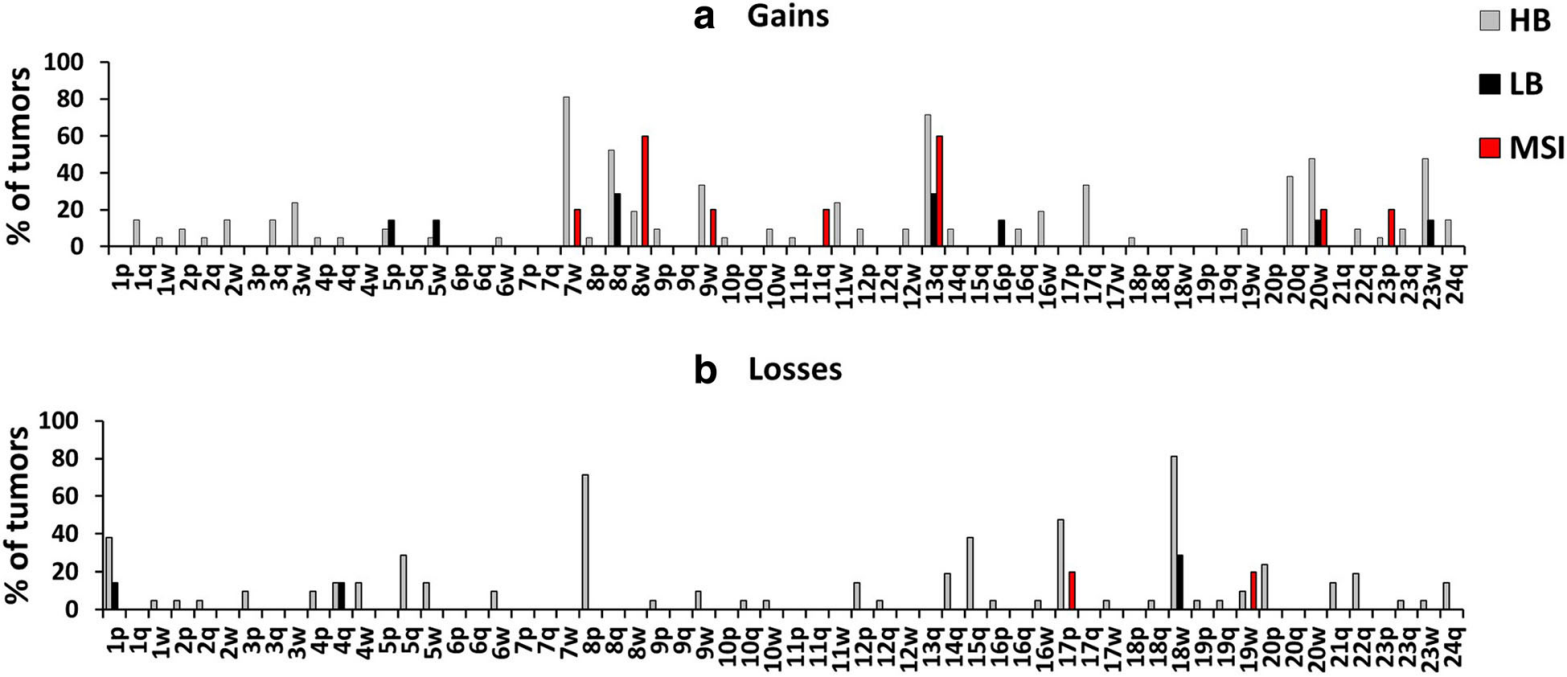
Broad gains (**a**) and losses (**b**) in 21 HB samples, 7 LB samples and 5 MSI samples from our series of CRCs. w: whole chromosome, p: short arm, q: long arm

Apart from 16p, whose gains have been found only in the LB group, all chromosomal gains found in LB CRCs were also found in the HB group (Fig. 1). This was also valid for losses: chromosomes affected in the LB group were the same chromosomes commonly affected in the HB group (Fig. 1). Gains of whole chromosome 8 (8w) were more common among MSI samples compared to HB and LB tumors (Fig. 1). In Fig. 1 data obtained in our series of MSI tumors are shown. Indeed, broad gains of chromosomes 7, 8, 9, 13, 20 were also observed by Sveen et al. in a different series of 27 MSI samples [15]. Loss of 18w was far less frequent in LB and MSI tumors of our series, as well as in the MSI CRCs studied by Sveen et al. [15]

### GI-based classification and histopathology

In the LB group, apart from 2 tumors of the rectum, 3/5 (60%) tumors were proximal (right colon) and 2/5 (40%) were distal (left colon and sigmoid colon). In the HB group, excluding 2 rectal tumors, 10/19 (53%) CRCs were proximal and 9/19 (47%) were distal. Of the 5 MSI samples of our series, 4/5 (80%) were proximal and only 1/5 (20%) was distal. Several CRC tumors showed mucinous histology or mucinous features. In the LB group 3/7 (43%) samples were mucinous adenocarcinomas (extracellular mucin > 50% of the tumor volume), 2/7 samples (29%) were adenocarcinomas with mucinous features (mucin 10–50%), 1/7 (14%) was a signet ring cell adenocarcinoma. In the MSI group 1/5 samples (20%) was a mucinous adenocarcinoma, 2/5 (40%) were adenocarcinomas with mucinous features. In the HB group 3/21 samples (14%) were mucinous adenocarcinomas, 2/21 (10%) were adenocarcinomas with mucinous features and 1/21 (5%) was a signet ring cell adenocarcinoma.

### Differentially expressed genes (DEGs) across GI-based CRC groups

Differences in gene expression between each CRC group and the normal colonic tissue were assessed. A fold change (FC) > 2 was chosen as a threshold for upregulated genes, whereas a FC < − 2 was chosen for downregulated genes. A false discovery rate (FDR) *p*-value < 0.05 was used to establish statistical significance [17]. In these expression studies, results regarding MSI tumors have been obtained using raw HTA data from 34 MSI samples provided by Sveen et al. (2017) [15].

Results on the top 30 differentially expressed genes (DEGs) are shown in Additional file 2: Figure S1 (upregulated in a, c and e; downregulated in b, d and f). As expected, markers of normal colonocytes, such as Solute Carrier Family 26 Member 3 (*SLC26A3*) [18, 19], Chloride Channel Accessory 4 (*CLCA4*) and Membrane Spanning 4-Domains A12 (*MS4A12*) [20, 21], as well as other enterocyte markers such as Carcinoembryonic Antigen Related Cell Adhesion Molecule 7 (*CEACAM7*) [22], Guanylate Cyclase Activator 2A (*GUCA2A*) [23], Aquaporin 8 (*AQP8*) [24], isoforms *CA1*, *CA2, CA4* of Carbonic Anhydrase and Keratin 20 (*KRT20*) [25, 26] were downregulated in the three CRC groups. Solute Carrier Family 12 Member 2 (*SLC12A2*), encoding the Na-K-Cl cotransporter isoform 1 (NKCC1), was the most highly upregulated transcript in the three CRC groups (first position in HB and third position in LB and MSI).

**Fig. 2:**
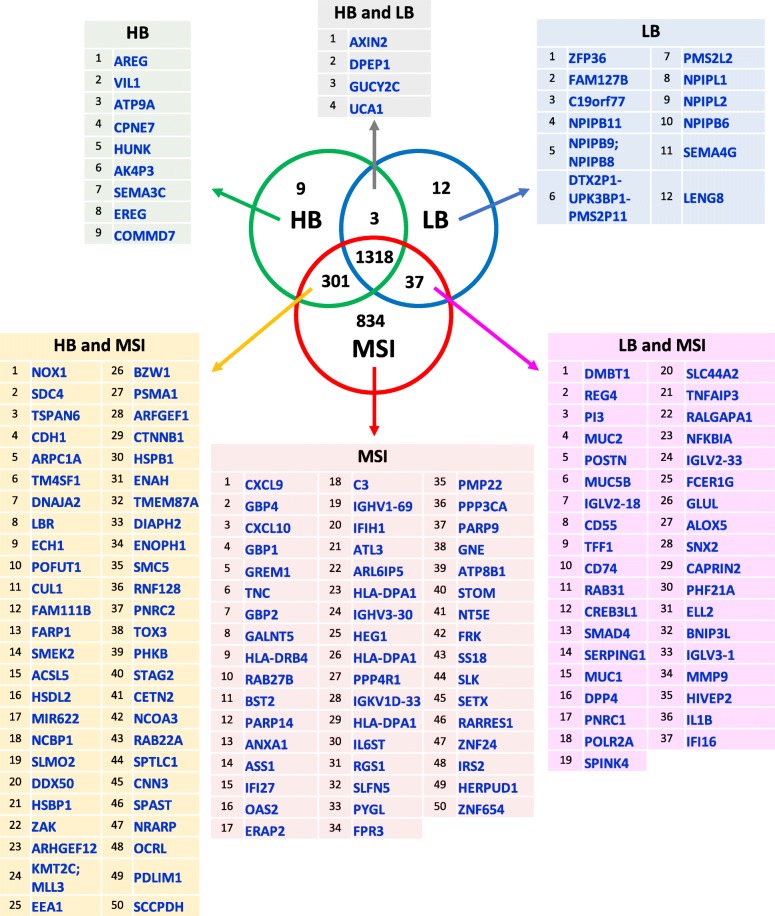
Specifically or shared upregulated genes across different CRC groups. Thresholds for fold-changes values and FDR adjusted-*p* values are reported in Table 1. Within each analyzed group or group intersection, the number of upregulated genes is reported. Top ranking upregulated genes (up to 50) were detailed for each group

**Fig. 3:**
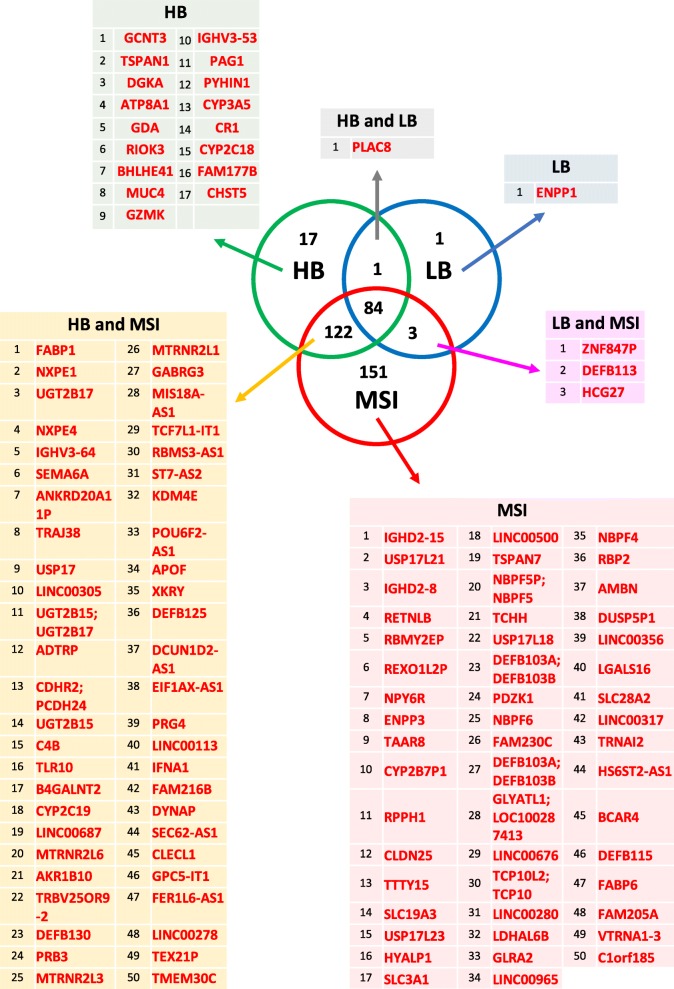
Specifically or shared downregulated genes across different CRC groups. Thresholds for fold-changes values and FDR adjusted-*p* values are reported in Table 2. Within each analyzed group or group intersection, the number of downregulated genes is reported. Top ranking downregulated DEGs (up to 50) were detailed for each group

In order to identify those DEGs that are specifically upregulated in each of the three CRC groups (specifically upregulated DEGs) or that are concurrently upregulated in two CRC groups (shared upregulated DEGs), we used the thresholds for FCs and FDRs reported in Table 1. A similar analysis (Table 2) was also performed for specifically or shared downregulated DEGs. Results are shown in Fig. 2 and Fig. 3.

**Table 1:** Thresholds for identification of specifically or shared upregulated DEGs

CRC groups			
	specifically upregulated DEGs		
	HB vs N	LB vs N	MSI vs N
HB	FC > 2 and FDR < 0.05	FC < 2 and FDR > 0.05	FC < 2 and FDR > 0.05
LB	FC < 2 and FDR > 0.05	FC > 2 and FDR < 0.05	FC < 2 and FDR > 0.05
MSI	FC < 2 and FDR > 0.05	FC < 2 and FDR > 0.05	FC > 2 and FDR < 0.05
	shared upregulated DEGs		
	HB vs N	LB vs N	MSI vs N
HB and LB	FC > 2 and FDR < 0.05	FC > 2 and FDR < 0.05	FC < 2 and FDR > 0.05
LB and MSI	FC < 2 and FDR > 0.05	FC > 2 and FDR < 0.05	FC > 2 and FDR < 0.05
HB and MSI	FC > 2 and FDR < 0.05	FC < 2 and FDR > 0.05	FC > 2 and FDR < 0.05
HB, LB and MSI	FC > 2 and FDR < 0.05	FC > 2 and FDR < 0.05	FC > 2 and FDR < 0.05

**Table 2:** Thresholds for identification of specifically or shared downregulated DEGs

CRC groups			
	specifically downregulated DEGs		
	HB vs N	LB vs N	MSI vs N
HB	FC < −2 and FDR < 0.05	FC > −2 and FDR > 0.05	FC > − 2 and FDR > 0.05
LB	FC > −2 and FDR > 0.05	FC < − 2 and FDR < 0.05	FC > − 2 and FDR > 0.05
MSI	FC > − 2 and FDR > 0.05	FC > − 2 and FDR > 0.05	FC < − 2 and FDR < 0.05
	shared downregulated DEGs		
	HB vs N	LB vs N	MSI vs N
HB and LB	FC < −2 and FDR < 0.05	FC < −2 and FDR < 0.05	FC > − 2 and FDR > 0.05
LB and MSI	FC > −2 and FDR > 0.05	FC < − 2 and FDR < 0.05	FC < − 2 and FDR < 0.05
HB and MSI	FC < − 2 and FDR < 0.05	FC > − 2 and FDR > 0.05	FC < − 2 and FDR < 0.05
HB, LB and MSI	FC < − 2 and FDR < 0.05	FC < − 2 and FDR < 0.05	FC < − 2 and FDR < 0.05

*FC* Linear Fold-change, *FDR* False Discovery Rate adjusted *p* value

**Fig. 4:**
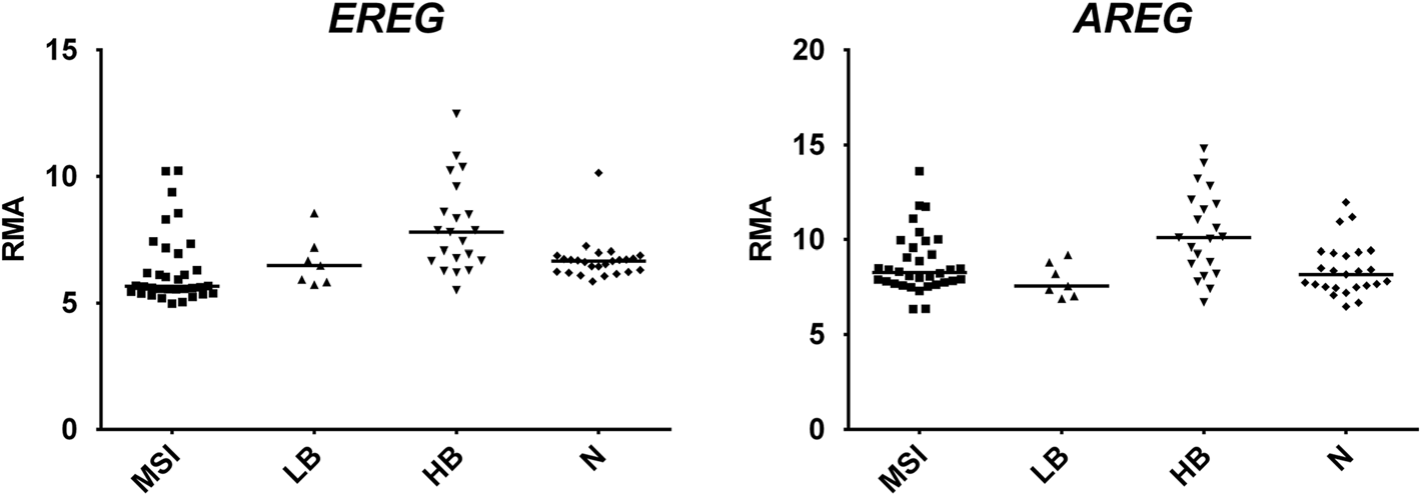
Dot plots of expression values of *AREG* and *EREG*, two upregulated DEGs in HB CRCs. Horizontal bars: median. *RMA*, Robust Multi-array Average. Statistical analysis was performed according to Benjamini–Hochberg [17]: *AREG*, HB vs N FDR-adjusted *p* = 0.002913, LB or MSI vs N: not significant; *EREG*, HB vs N FDR-adjusted *p* = 0.004002, LB or MSI vs N: not significant

HB tumors were characterized by upregulation of amphiregulin (*AREG*) and epiregulin (*EREG*), which are members of the epidermal growth factor (EGF) family, and have both been reported as upregulated in CRC [27–30] (Fig. 4). Moreover, a colonic epithelial marker such as Villin 1(*VIL1*) was specifically upregulated in HB tumors [31].

**Fig. 5:**
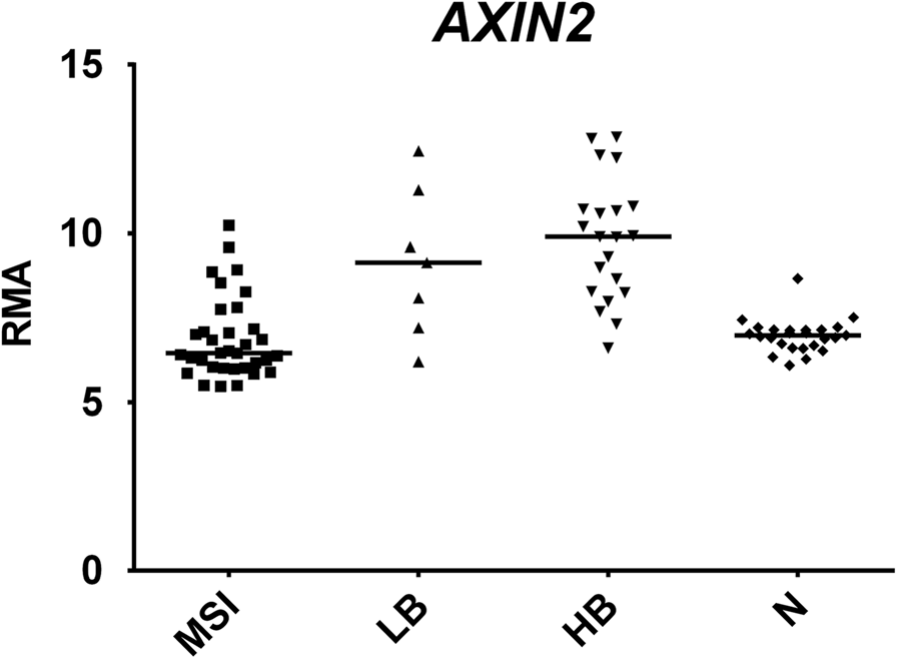
Dot plots of expression values of *AXIN2*, a representative DEGs upregulated in both HB and LB groups. Horizontal bars: median. *RMA*, Robust Multi-array Average. Statistical analysis was performed according to Benjamini–Hochberg [17]: HB vs N *p* = 7.53E-07, LB vs N *p* = 0.005866; MSI vs N not significant

Both LB and, at a major extent, HB tumors overexpressed *AXIN2*, a negative feedback inhibitor of Wnt/β-catenin pathway [32] whose upregulation accompanies WNT signaling activation [33] (Fig. 5). The stem-cell marker OLFM4 was significantly upregulated in all CRC subgroups, but FC values were much higher in HB and LB (26- and 74-fold increase respectively) in comparison to MSI tumors (3.5-fold increase).

Analysis of the list of specifically upregulated genes in the MSI group by the “Functional Annotation Tools” of DAVID Bioinformatics Resources 6.8 [34] reveals a significant 3.66-fold enrichment of the term “immunity” (FDR = 6.36E-18), a 9.68-fold enrichment of the term “Immunoglobulin V region” (FDR = 1.57E-9), and a 4.35-fold enrichment of the term “regulation of immune response” (FDR = 6.69E-9), in agreement with the well-known increase in immune cells infiltrate in such CRC group [35]. The list of specifically downregulated genes in MSI tumors shows a significant enrichment in genes related to protein deubiquitation (members of the ubiquitin specific peptidase 17-like family; fold-enrichment: 17.8; FDR = 3.82E-4) and to the family of microbicidal and cytotoxic peptides defensin (DEFA1, DEFA1B, DEFB103A, DEFB103B, DEFB115, DEFB121, DEFB136; fold-enrichment: 25.65; FDR = 3.42E-4).

**Fig. 6:**
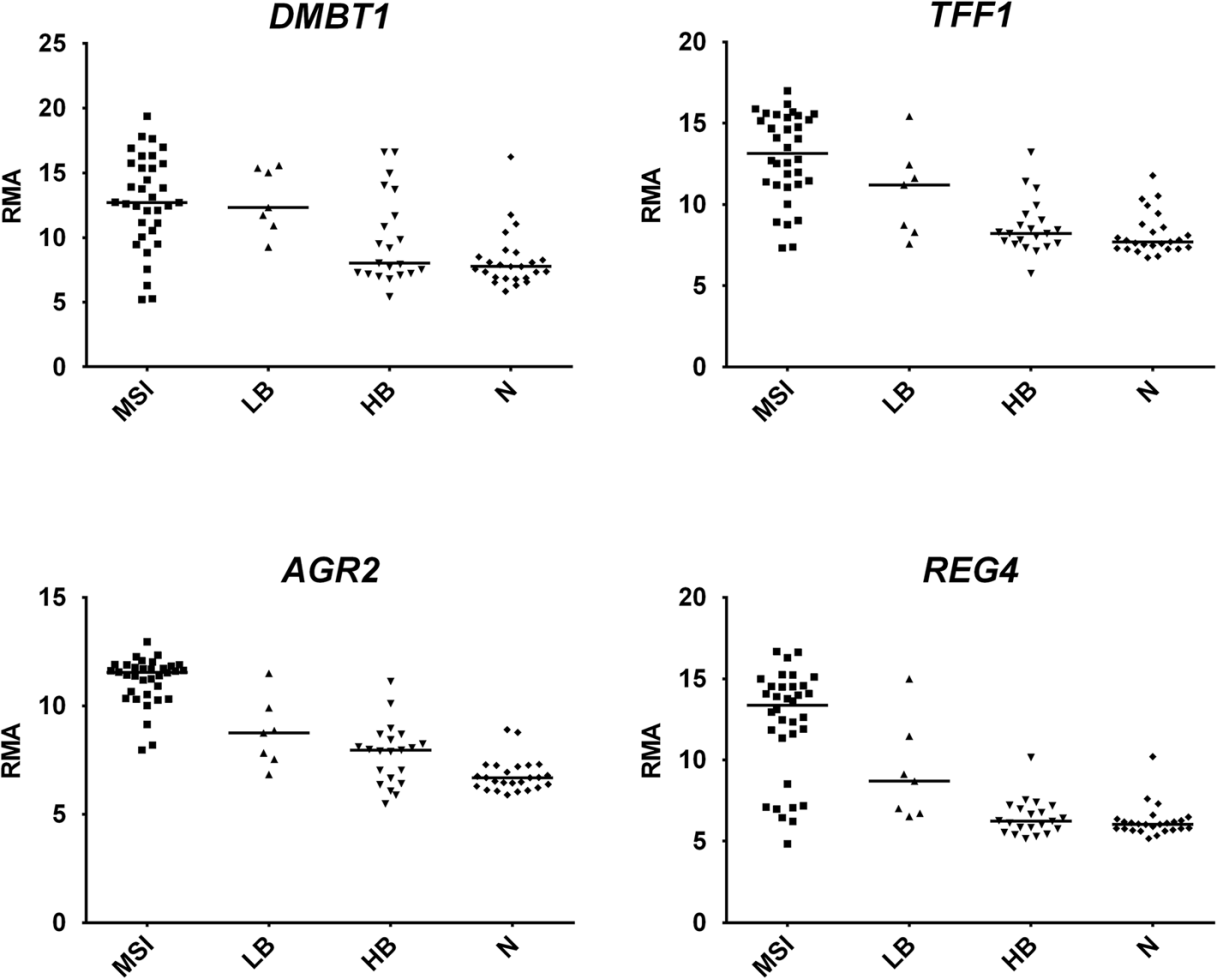
Dot plots of DEGs upregulated in both LB and MSI groups. Horizontal bars: median. *RMA*, Robust Multi-array Average. Statistical analysis was performed according to Benjamini–Hochberg [17]: *DMBT1*, HB vs N not significant, LB vs N FDR-adjusted *p* = 0.003952, MSI vs N FDR-adjusted *p* = 4.60E-07; *TFF1,* HB vs N not significant, LB vs N FDR-adjusted *p* = 0.020418, MSI vs N FDR-adjusted *p* = 1.75E-11; *MUC2*, HB vs N not significant, LB vs N FDR-adjusted *p* = 0.041522, MSI vs N FDR-adjusted *p* = 0.001072; *REG4,* HB vs N not significant, LB vs N FDR-adjusted *p* = 0.00027, MSI vs N FDR-adjusted *p* = 5.31E-12

Interestingly, LB and MSI tumors shared upregulation of genes typical for secretory cells, such as Mucin 2 (*MUC2*), Mucin 1 (*MUC1*), Mucin 5B (*MUC5B*), Trefoil Factor 1 (*TFF1*), Deleted In Malignant Brain Tumors 1 (*DMBT1*)*,* Regenerating Family Member 4 (*REG4*), although upregulation is generally higher for MSI tumors (Fig. 6). Using the “Functional Annotation Tools” of DAVID Bioinformatics Resources 6.8 [34], the list of shared upregulated genes in LB and MSI groups shows a fold-enrichment of 4.24 (FDR = 0.00286) of the category term “secreted” in the UP_KEYWORDS (genes: MUC1, MUC2, REG4, MMP9, SPINK4, POSTN, SERPING1, IGLV3–1, CD55, PI3, IL1B, TFF1, MUC5B, DPP4, DMBT1). LB and MSI tumors also showed upregulation of *POSTN* (Periostin), a gene involved in various signaling pathways and extracellular matrix (ECM) remodeling [36, 37].

**Fig. 7:**
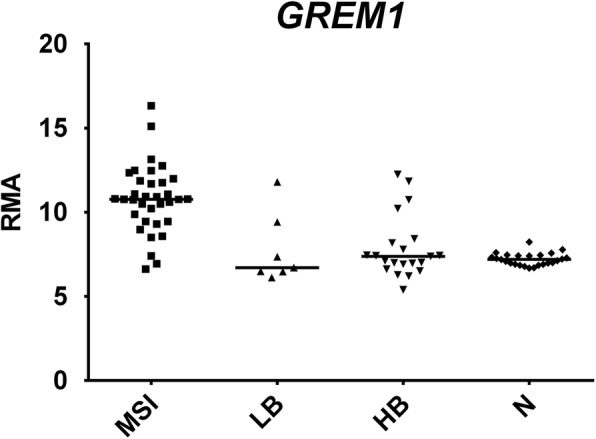
Dot plots of expression values of *GREM1*, a representative DEGs upregulated in MSI groups. *RMA*, Robust Multi-array Average. Statistical analysis was performed according to Benjamini–Hochberg, [17]: HB vs N not significant; LB vs N not significant, MSI vs N FDR-adjusted *p* = 3.94E-12

As shown in Fig. 7, MSI tumors showed a specific upregulation of bone morphogenetic protein antagonist Gremlin 1 *(GREM1*). GREM1 encodes a protein whose expression is weak or absent in normal colorectal epithelium but increases in CRCs, especially those with serrated histology and low tumor stage [38]. A 40-kb duplication of a region upstream of GREM1 has been associated with hereditary mixed polyposis syndrome (HMPS) [39], and a 16-kb duplication in the regulatory region of GREM1 has been found as the disease-causing genetic alteration in a family with attenuated/atypical polyposis syndrome [40]. Gain-of function mutations in GREM1 impair BMP signaling, which normally exerts a negative control on intestinal epithelial cells [41]. Moreover, GREM1 epithelial expression has been shown to contribute to colonic carcinogenesis by reconferring stem-cell features to progenitor cells located at a distance from the stem-cell niche [42]. Finally, it has been demonstrated in glioblastomas that cancer stem cells express GREM1 to counteract BMP-driven differentiation, and promote their self-renewal [43].

### Consensus molecular subtypes (CMS)

We used the Single Sample Predictor (SSP) for CMS classification of HB, LB, and MSI samples (Table 3). About 80% (27/34) of the MSI samples were attributed the CMS1 subtype, as expected, since the CMS1 subtype has been defined as “MSI-Immune”, and most of its samples are MSI [12]. 76% (16/21) of the HB tumors were classified as CMS2, which is indeed the subtype with the highest number of somatic CNAs [12]. The LB group was instead heterogeneous with respect to the proportion of CMS subtypes: 43% of the samples (3/7) could not be attributed to any of the 4 CMS classes, whereas 29% (2/7) were classified as CMS3, 14% (1/7) as CMS1 and 14% (1/7) as CMS2.

**Table 3:** Single sample predictor (SSP) results

CMS^a^ subtype	HB^b^ (*n* = 21)	LB^c^ (*n* = 7)	MSI^d^ (*n* = 34)
CMS1	0%	14% (*n* = 1)	79% (*n* = 27)
CMS2	76% (*n* = 16)	14% (*n* = 1)	0% (*n* = 0)
CMS3	5% (*n* = 1)	29% (*n* = 2)	12% (*n* = 4)
CMS4	5% (*n* = 1)	0% (*n* = 0)	0% (*n* = 0)
NA	14% (*n* = 3)	43% (*n* = 3)	9% (*n* = 3)

Percentage of CMS1, CMS2, CMS3, CMS4 and NA (not classified) samples in the HB, LB and MSI groups. ^a^CMS, Consensus Molecular Subtypes; ^b^HB, High-BCNA tumor samples; ^c^LB, Low-BCNA tumors samples; ^d^MSI, Microsatellite-instable tumor samples

## Discussion

Subdividing MSS CRC samples according to their BCNA scores lead to the identification of two MSS tumor groups, LB (with lower numbers of BCNAs) and HB (with higher numbers of BCNAs), which differ for histopathology and gene expression profile.

HB tumors showed upregulation of the epithelial marker *VIL1* and the EGFR ligands *AREG* and *EREG*. *AREG* and/or *EREG* overexpression is frequent in CRC, and is inversely correlated to promoter methylation [27, 28, 30]. *AREG*/*EREG* overexpression and activation of the EGF pathway is a feature of CIN-positive CRCs, especially for carcinomas of the distal colon [28, 29]. Of note, *AREG* and *EREG* overexpression is predictive of response to cetuximab as well as other EGFR blockade agents [44–46].

The LB group and the MSI group – both characterized by low numbers of BCNAs - showed similarities in the pattern of upregulated genes involved in secretory processes (*MUC2*, *MUC5B*, *TFF1*, *DMBT1*, *REG4*, *POSTN*).

MUC2 is the most abundant mucin in gastrointestinal mucus and its expression is generally reduced in CRC, except for mucinous CRCs, which preserve MUC2 expression [47, 48]. Trefoil factor TFF1 is also secreted by gastrointestinal mucus-producing cells, preferentially in normal stomach and Brunner glands, contributing to mucus stabilization and mucosal protection [49]. *MUC5B* was indeed overexpressed in MSI samples, as previously observed at protein level by Walsh et al. (2013) [50].

Overexpression of the above-mentioned mucus-related genes in the LB and MSI groups compared to the HB one was concordant with histopathology data, since 86% LB samples showed mucin production, whether extracellular or intracellular. In particular, 43% were mucinous adenocarcinomas, 29% adenocarcinomas with mucinous features and 14% signet ring cell adenocarcinomas. We did not have information on the histopathology of the 34 MSI samples from Sveen et al. (2017) [15], but 20% of our MSI samples were mucinous adenocarcinomas and 40% were adenocarcinomas with mucinous features. Mucinous CRCs are known to be enriched in MSI tumors [51, 52] and to harbor a reduced number of copy number aberrations [9]. In contrast, in the HB group only 14% samples were mucinous adenocarcinomas, 10% were adenocarcinomas with mucinous features and 5% signet ring cell adenocarcinomas. Moreover, other genes upregulated in both MSI and LB, such as *REG4* and *CD55* had already been described as upregulated in mucinous MSI tumors compared to normal colonic tissue [53].

*REG4* is a protein with anti-apoptotic and secretory functions [54], which appears to be a marker of a subset of neuroendocrine intestinal cells [55]. *REG4* expression is elevated in mucinous CRCs, in concert with high MUC2 expression [56]. *REG4* is also a marker of the deep crypt secretory (DCS) cells, which are mucous-type cells intercalated with LGR5^+^ base columnar stem cells of colonic crypts [57], and play a role as Paneth cell equivalents for the stem cell niche of the colon [58]. DCS cells also overexpress *AGR2* [59].

In the present study, we found that a subset of genes usually upregulated in MSI tumors is also upregulated in MSS LB tumors, not only in comparison to the normal colonic tissue, but also compared to MSS HB tumors. In conclusion, the LB and MSI groups appear to be characterized by specific genes involved in secretory processes, colon mucus barrier, and mucosal protection, whereas HB tumors show overexpression of a subset of genes typical for surface colonocytes, along with EGF signaling agonists *AREG* and *EREG*, whose upregulation might be of predictive relevance for therapeutic choices.

Our results confirm and extend data obtained by Hugen et al. [9], who analyzed their own patient cohort and TCGA level 3 SNP6 data, and found a reduced rate of copy number aberrations in mucinous adenocarcinomas. In the present study we show that selecting CRC tumors according to low BCNA scores it is possible to obtain a group enriched both in mucinous adenocarcinomas and in adenocarcinomas with mucinous features. Such conclusion was strengthened by our data showing an increased gene expression of mucinous markers in LB tumors. On this regard, it is relevant that a large fraction of LB tumors cannot be classified in any of the Consensus Molecular Subtypes and *BCNA number* may represent a useful parameter to provide additional biological information on this group of tumors.

Davoli et al. (2017) [60] reported that tumors with high levels of arm and whole-chromosome somatic copy number aberrations (SCNA), largely corresponding to our high BCNA scores, showed a reduced expression of markers for cytotoxic immune cell infiltrates. These authors suggest that SCNA levels are a stronger predictor of markers of cytotoxic immune cell infiltration than tumor mutational load and report that in melanoma the combination of the tumor SCNA score and the tumor mutational load was a better predictor of survival after immunotherapy than either biomarker alone. Our data add further information on the possibility to use a quantitative index of arm or whole-chromosome SCNAs (or BCNAs) in order to predict relevant biological features of the tumors. Aneuploidy is the classical term used to indicate the presence of broad somatic copy number alterations, and Taylor et al. (2018) [61] recently proposed a so-called “aneuploidy score” based on the total number of arm-level events in each tumor. They confirmed that such aneuploidy score correlates with cell-cycle genes and anticorrelates with immune levels. In the present paper, we suggest that, in the context of CRC, BCNA or aneuploidy scores can also be correlated to other biological features, such as the presence of a mucinous component. Mucinous adenocarcinoma and signet-ring cell carcinoma, CRCs that produce excess mucin, account for 10–15% and 0.1–2.4% of CRC cases, respectively. The poor prognosis of signet-ring cell carcinoma has been widely reported, while the prognosis of mucinous adenocarcinoma remains controversial [62]. The combined use of BCNA score, MSI status and mucin gene expression profile could provide a better molecular characterization tool to solve this long-standing issue.

## Conclusions

In the present paper we show that the number of BCNAs, evaluated by genome-wide techniques, identifies tumor groups differing in their expression profile and histopathological features. Therefore, its routine use in the classification of CRC samples, along with MSI testing and detection of sequence variants, provides additional biological information. Since BCNA score is a reliable parameter with potential prognostic and/or predictive value, it should be kept in consideration in studies aimed to evaluate drug response in CRC subgroups.

## Additional files

Additional file 1: **Table S1.** Clinicopathological data. (DOCX 196 kb)

Additional file 2: **Figure. S1** Top 30 differentially expressed genes between CRC groups and normal tissue (this number was chosen only for display purposes). *Left panels*: genes upregulated in HB (a), LB (c) and MSI CRCs (e) compared to normal colonic tissue. *Right panels*: genes downregulated in HB (b), LB (d) and MSI CRCs (f) compared to normal colonic tissue. Black columns show results in tumor samples, white columns in normal colonic tissue. Upregulated genes are listed in descending order according to fold change in gene expression compared to normal tissue, whereas downregulated genes are listed in ascending order. *RMA*, Robust Multi-array Average. *CA2* gene is downregulated in the MSI group as well, but it is not displayed in this figure because it ranks 34th in the list of downregulated genes in MSI tumors compared to the normal tissue. (DOCX 17 kb)

## Abbreviations

BCNA: Broad Copy Number Aberration
CMS: Consensus molecular subtypes
CRC: ColoRectal Cancer
HB: Higher numbers of BCNAs
HMPS: Hereditary mixed polyposis syndrome
HTA: Whole-transcript expression analysis by Human Transcriptome Array
LB: Lower numbers of BCNAs
MSI: MicroSatellite-Instable
MSS: MicroSatellite-Stable
SSP: Single sample predictor
