# Supplementary material for: Gene expression profiles in genome instability-based classes of colorectal cancer
Source: BMC Cancer. 2018 Dec 18;18:1265. doi: 10.1186/s12885-018-5174-z (PMC6299572; doi:10.1186/s12885-018-5174-z)
Supplement: Supplementary file 1 — Table S1. Clinicopathological data. (DOCX 196 kb) [file 12885_2018_5174_MOESM1_ESM.docx]

**Additional file 1: Table S1** Clinicopathological data.

| **Sample name** | **Anatomical site** | **Histology** | **Tumor stage (AJCC)** | |
| --- | --- | --- | --- | --- |
| **HB** | | | |  |
| 1 | sigmoid colon | adenocarcinoma | stage 4 | |
| 2 | left colon | adenocarcinoma | stage 3 | |
| 3 | right colon | adenocarcinoma | stage 3 | |
| 4 | sigmoid colon | adenocarcinoma | stage 2 | |
| 5 | right colon | adenocarcinoma | stage 2 | |
| 6 | right colon | adenocarcinoma | stage 4 | |
| 7 | left colon | mucinous adenocarcinoma | stage 3 | |
| 8 | left colon | mucinous adenocarcinoma | stage 3 | |
| 9 | right colon | adenocarcinoma | stage 4 | |
| 10 | sigmoid colon | adenocarcinoma | stage 2 | |
| 11 | left colon | adenocarcinoma | stage 2 | |
| 12 | right colon | adenocarcinoma | stage 2 | |
| 13 | right colon | adenocarcinoma with mucinous features | stage 3 | |
| 14 | right colon | adenocarcinoma | stage 3 | |
| 15 | right colon | mucinous adenocarcinoma | stage 3 | |
| 16 | rectum | adenocarcinoma with mucinous features | stage 2 | |
| 17 | right colon | adenocarcinoma | stage 2 | |
| 18 | right colon | signet-ring cell adenocarcinoma | stage 3 | |
| 19 | rectum | adenocarcinoma | stage 3 | |
| 20 | sigmoid colon | adenocarcinoma | stage 3 | |
| 21 | sigmoid colon | adenocarcinoma | stage 2 | |
| **LB** | | | |  |
| 22 | rectum | adenocarcinoma | stage 4 | |
| 23 | left colon | mucinous adenocarcinoma | stage 2 | |
| 24 | left colon | mucinous adenocarcinoma | stage 2 | |
| 25 | rectum | mucinous adenocarcinoma | stage 2 | |
| 26 | right colon | adenocarcinoma with mucinous features | stage 2 | |
| 27 | right colon | signet-ring cell adenocarcinoma | stage 3 | |
| 28 | right colon | adenocarcinoma with mucinous features | stage 3 | |
| **MSI** | | | |  |
| 29 | right colon | adenocarcinoma with mucinous features | stage 3 | |
| 30 | left colon | adenocarcinoma with mucinous features | stage 3 | |
| 31 | right colon | mucinous adenocarcinoma | stage 3 | |
| 32 | right colon | adenocarcinoma | stage 3 | |
| 33 | right colon | adenocarcinoma | stage 2 | |

Age at surgery (average + SD): 65.7 + 15.3 (HB), 77.4 + 6.5 (LB), 65.4 + 21 (MSI).

% of males: 52% (HB), 57% (LB), 80% (MSI)
