# Supplementary material for: Gene expression profiles in genome instability-based classes of colorectal cancer
Source: BMC Cancer. 2018 Dec 18;18:1265. doi: 10.1186/s12885-018-5174-z (PMC6299572; doi:10.1186/s12885-018-5174-z)
Supplement: Supplementary file 2 — Figure. S1 Top 30 differentially expressed genes between CRC groups and normal tissue (this number was chosen only for display purposes). Left panels: genes upregulated in HB (a), LB (c) and MSI CRCs (e) compared to normal colonic tissue. Right panels: genes downregulated in HB (b), LB (d) and MSI CRCs (f) compared to normal colonic tissue. Black columns show results in tumor samples, white columns in normal colonic tissue. Upregulated genes are listed in descending order according to fold change in gene expression compared to normal tissue, whereas downregulated genes are listed in ascending order. RMA, Robust Multi-array Average. CA2 gene is downregulated in the MSI group as well, but it is not displayed in this figure because it ranks 34th in the list of downregulated genes in MSI tumors compared to the normal tissue. (DOCX 17 kb) [file 12885_2018_5174_MOESM2_ESM.docx]

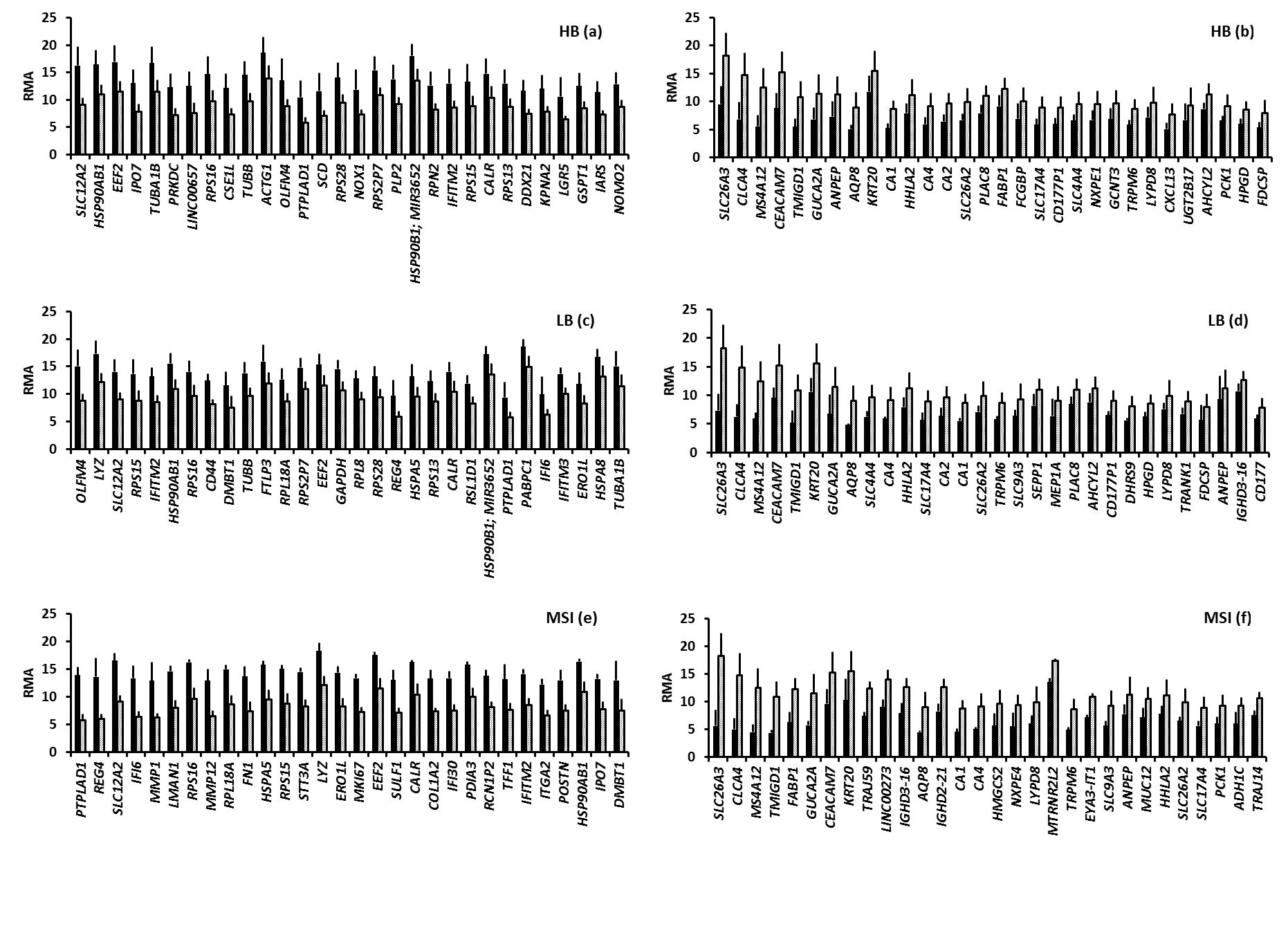
 **Additional file 2: Figure S1** Top 30 differentially expressed genes between CRC groups and normal tissue (this number was chosen only for display purposes). *Left panels*: genes upregulated in HB (a), LB (c) and MSI CRCs (e) compared to normal colonic tissue. *Right panels*: genes downregulated in HB (b), LB (d) and MSI CRCs (f) compared to normal colonic tissue. Black columns show results in tumor samples, white columns in normal colonic tissue. Upregulated genes are listed in descending order according to fold change in gene expression compared to normal tissue, whereas downregulated genes are listed in ascending order. *RMA*, Robust Multi-array Average. *CA2* gene is downregulated in the MSI group as well, but it is not displayed in this figure because it ranks 34^th^ in the list of downregulated genes in MSI tumors compared to the normal tissue.
